# Supplementary material for: Radioactive Seed Localization for Nonpalpable Breast Lesions: Systematic Review and Meta-Analysis
Source: Diagnostics (Basel). 2024 Feb 17;14(4):441. doi: 10.3390/diagnostics14040441 (PMC10887864; doi:10.3390/diagnostics14040441)
Supplement: Supplementary file 1 [file diagnostics-14-00441-s001.zip › diagnostics-2741586-supplementary.pdf]

Supporting Information for

Radioactive seed localization for nonpalpable breast lesions: systematic review and meta-analysis

1. Search strategy

1. ClinicalTrials.gov

   ((breast cancer*) OR (breast neoplasm*) OR (breast carcinoma*) OR (breast lesion*) OR ("nonpalpable") OR ("non-palpable") OR ("non palpable") OR ("impalpable") OR ("occult") OR (breast surgery) OR (radioguided surgery) OR (lumpectomy*)) AND ((localization*) OR (radioactive seed*) OR (radioguided seed*) OR (iodine seed*) OR (iodine-125) OR (125i seed*) OR (rollis) OR (rsl) OR (wire localization) OR (wgl) OR (radioguided occult lesion localization) OR (roll))

2. Embase

   #1. 'breast cancer'/exp OR 'breast surgery'/exp

   #2. 'breast cancer*' OR 'breast neoplasm*' OR 'breast carcinoma*' OR 'breast lesion*'

   #3. 'nonpalpable' OR 'non-palpable' OR 'non palpable' OR 'impalpable' OR 'occult'

   #4. 'breast surgery' OR 'radioguided surgery' OR 'lumpectomy*'

   #5. 'radioactive seed'/exp

   #6. 'seed* localization*' OR 'radioactive seed*' OR 'radioguided seed*' OR 'iodine seed*' OR 'iodine-125' OR '125i seed*' OR 'rollis' OR 'rsl'

   #7. 'wire localization' OR 'wgl' OR 'radioguided occult lesion localization' OR 'roll'

   #8. #1 OR #2 OR #3 OR #4

   #9. #5 OR #6 OR #7

   #10. #8 AND #9

3. LILACS
   #1 (mh:(breast cancer)) OR (mh:(breast surgery))
   #2 (breast cancer*) OR (breast neoplasm*) OR (breast carcinoma*) OR (breast lesion*)
   #3 (nonpalpable) OR (non-palpable) OR (non palpable) OR (impalpable) OR (occult)
   #4 (breast surgery) OR (radioguided surgery) OR (lumpectomy*) 2394
   #5 #1 OR #2 OR #3 OR #4
   #6 (mh:(localization))
   #7 (localization*) OR (radioactive seed*) OR (radioguided seed*) OR (iodine seed*) OR (iodine-125) OR (125I seed*) OR (rollis) OR (rsl) 2343
   #8 (wire localization) OR (wgl) OR (radioguided occult lesion localization) OR (roll)
   #9 #6 OR #7 OR #8
   #10 #5 AND #9

4. PubMed
   (((((breast cancer[MeSH Terms]) OR (breast surgery[MeSH Terms])) OR ((((breast cancer*) OR (breast neoplasm*)) OR (breast carcinoma*)) OR (breast lesion*))) OR ((((("nonpalpable") OR ("non palpable")) OR ("non-palpable")) OR ("impalpable")) OR ("occult"))) OR (((breast surgery) OR (radioguided surgery)) OR (lumpectomy*))) AND (((radioactive seed[MeSH Terms]) OR ((((((((seed* localization*) OR (radioactive seed*)) OR (radioguided seed*)) OR (iodine seed*)) OR (iodine-125)) OR (125I seed*)) OR (rollis)) OR (rsl))) OR ((((wire localization) OR (wgl)) OR (radioguided occult lesionlocalization)) OR (roll)))

5. SciELO
   #1 (breast cancer*) OR (breast neoplasm*) OR (breast carcinoma*) OR (breast lesion*)
   #2 ("nonpalpable") OR ("non-palpable") OR ("non palpable") OR ("impalpable") OR ("occult")
   #3 (breast surgery) OR (radioguided surgery) OR (lumpectomy*) 756

#4 #1 OR #2 OR #3

#5 (localization*) OR (radioactive seed*) OR (radioguided seed*) OR (iodine seed*) OR (iodine-125) OR (125i seed*) OR (rollis) OR (rsl)

#6 (wire localization) OR (wgl) OR (radioguided occult lesion localization) OR (roll)

#7 #5 OR #6

#8 #4 AND #7

6. Web of Science

   #1 ALL= (breast cancer*) OR ALL= (breast neoplasm*) OR ALL= (breast carcinoma*) OR ALL = (breast lesion*)

   #2 ALL= ("nonpalpable") OR ALL= ("non-palpable") OR ALL= ("non palpable") OR ALL= ("impalpable") OR ALL= ("occult")

   #3 ALL= (breast surgery) OR ALL= (radioguided surgery) OR ALL= (lumpectomy*)

   #4 #1 OR #2 OR #3

   #5 ALL= (seed localization) OR ALL= (radioactive seed*) OR ALL= (radioguided seed*) OR ALL= (iodine seed*) OR ALL= (iodine-125) OR ALL= (125i seed*) OR ALL= (rollis) OR ALL= (rsl)

   #6 ALL= (wire localization) OR ALL= (wgl) OR ALL= (radioguided occult lesion localization) OR ALL= (roll)

   #7 #5 OR #6

   #8 #4 AND #7

7. Manual search in reference lists
   a. Ahmed, M.; Douek, M. Radioactive seed localisation (RSL) in the treatment of non-palpable breast cancers: Systematic review and meta-analysis. The Breast, v. 22, n. 4, p. 383-388, 2013.
   b. Barentsz, M. W.; Van Den Bosch, M. A. A. J.; Veldhuis, W. B.; et al. Radioactive seed localization for non-palpable breast cancer. Br J Surg., v. 100, n. 5, p. 582-588, 2013.

c. Chan, B. K. Y.; Wiseberg-Firtell, J. A.; Jois, R. H. S.; et al. Localization techniques for guided surgical excision of non-palpable breast lesions. Cochrane Database of Systematic Reviews, v. x, n. 12, p. x-x, 2015.
d. Gray, R. J.; Pockaj, B. A.; Garvey, E.; et al. Intraoperative margin management in breast-conserving surgery: a systematic review of the literature. Ann Surg Oncol., v. 25, n. 1, p. 18-27, 2018.
e. Fusco, R.; Petrillo, A.; Catalano, O.; et al. Procedures for location of non-palpable breast lesions: a systematic review for the radiologist. Breast Cancer, v. 21, n. 5, p. 522–531, 2014.
f. Moreira, I. C.; Ventura, S. R.; Ramos, I.; et al. Preoperative localisation techniques in breast conservative surgery: A systematic review and meta-analysis. Surgical Oncology, v. 35, n. x, p. 351–373, 2020.
g. Pouw, B.; Wit-Van Der Veen, L. J.; Stokkel, M. P. M.; et al. Heading Toward Radioactive Seed Localization in Non-Palpable Breast Cancer Surgery? A Meta-Analysis. Journal of Surgical Oncology, v. 111, n. 2, p.185–191, 2015.
h. Wang, G.; Tsikouras, P.; Zuo, H.; et al. Radioactive seed localization and wire guided localization in breast cancer: A systematic review and meta-analysis. JBUON, v. 24, n. 1, p. 48-60, 2019.
i. Xu, X. J.; Li, J. J.; Ji, W. B. An updated Meta-Analysis of radioactive seed localization versus wire-guided localization in the treatment of nonpalpable breast lesions. Breast J., v. 24, n. 4, p. 673-675, 2018.
j. Aetna. Breast Biopsy Procedures. Medical Clinical Policy Bulletins, 2020.
k. Preoperative seed Placement for breast cancer surgery: clinical effectiveness, cost-efectiveness, and guidelines. Ottawa: CADTH, 2019
l. INESSS. Utilization de la bille radioactive pour la localisation preoperatoire de tumeurs mammaires non palpables. Note informative, 2016.

2. Eligibility criteria of studies for review

- **Population**

Inclusion: women, of any age over eighteen years old, diagnosed with a nonpalpable breast lesion, eligible for surgical intervention.
Exclusion: age under 18, pregnancy, men.

- **Intervention**

Inclusion: RSL with Iodine-125 for nonpalpable breast lesion conservative surgery.
Exclusion: RSL for other regions than the breast.

- **Comparator**

Inclusion: WGL and ROLL for nonpalpable breast lesion conservative surgery.
Exclusion: WGL and ROLL for other regions than the breast.

- **Outcomes**

Primaries: surgical margins, intraoperative re-excision, reoperation, recurrence, complications.
Secondaries: specimen volume, SLNB identification failure, localization time, operation time, interval time between localization and surgery, intervention evaluation by the patient and medical team.

- **Study design**

Inclusion: randomized clinical trial and cohort study with more than 10 patients, who compare RSL against ROLL and/or WGL.
Exclusion: reviews, letters, abstracts, comments, in vitro assays.

3. Summary of the Results

Figure SI1 summarizes the results.

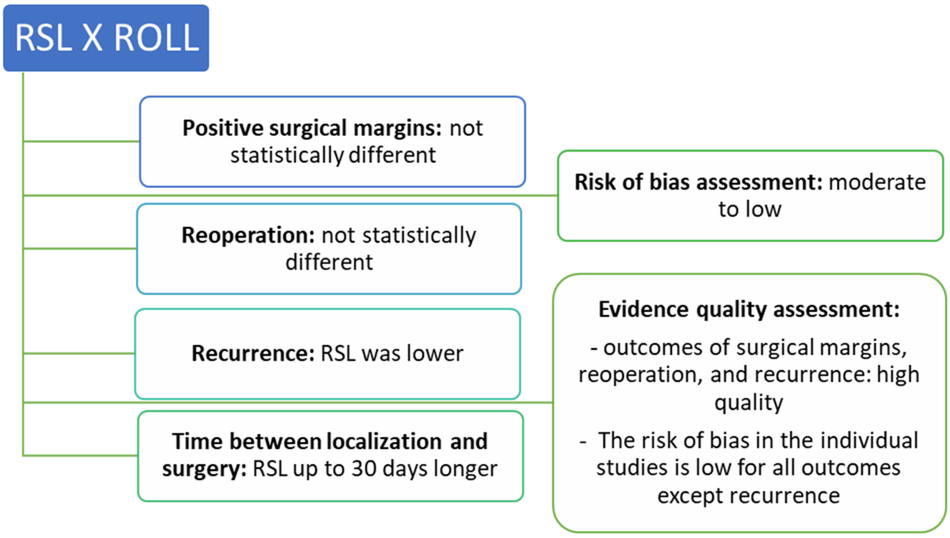

a)

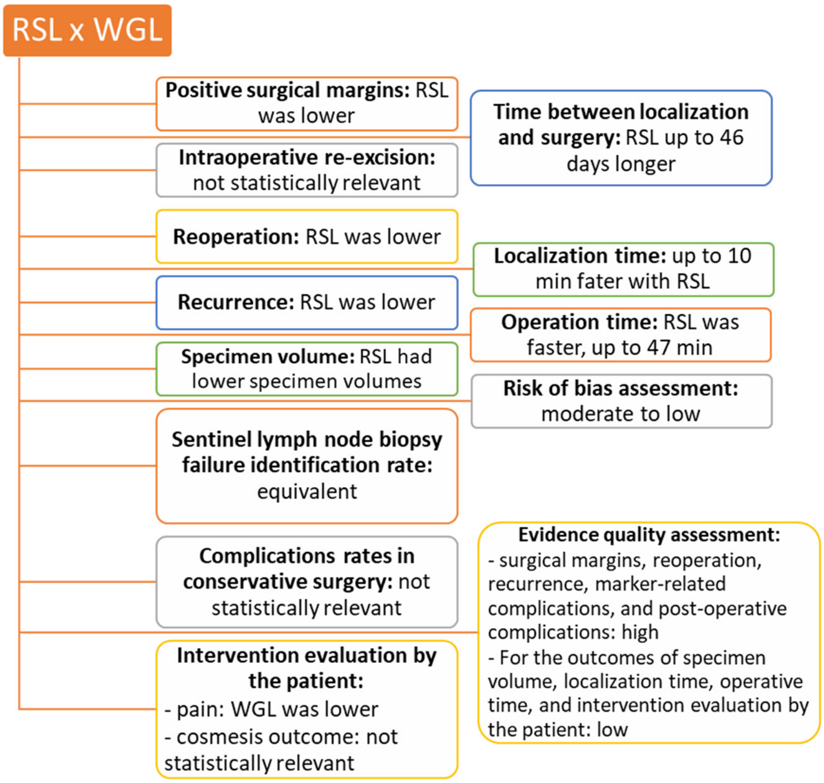

b)

Figure SI1: Summary of the results a) RSL x ROLL b) RSL x WGL.

4. Observations

- The outcome of the intervention evaluation by the medical team was reported by only 1 study and therefore data synthesis was not performed. Outcomes of positive surgical margins, intraoperative re-excision, reoperation, recurrence, specimen volume, sentinel node biopsy identification failure, complications, intervention evaluation by the patient, time between localization and surgery, localization time, and operative time are presented in the subtopics below.
